# Hyponatremia as a Predictor of Mortality in Peritoneal Dialysis Patients

**DOI:** 10.1371/journal.pone.0111373

**Published:** 2014-10-29

**Authors:** Tae Ik Chang, Yung Ly Kim, Hyungwoo Kim, Geun Woo Ryu, Ea Wha Kang, Jung Tak Park, Tae-Hyun Yoo, Sug Kyun Shin, Shin-Wook Kang, Kyu Hun Choi, Dae Suk Han, Seung Hyeok Han

**Affiliations:** 1 Department of Internal Medicine, NHIS Medical Center, Ilsan Hospital, Goyangshi, Gyeonggi–do, Republic of Korea; 2 Department of Internal Medicine, College of Medicine, Yonsei University, Seoul, Republic of Korea; 3 Brain Korea 21 for Medical Science, Severance Biomedical Science Institute, Yonsei University, Seoul, Republic of Korea; Sao Paulo State University, Brazil

## Abstract

**Background and Aim:**

Hyponatremia is common in patients with chronic kidney disease and is associated with increased mortality in hemodialysis patients. However, few studies have addressed this issue in peritoneal dialysis (PD) patients.

**Methods:**

This prospective observational study included a total of 441 incident patients who started PD between January 2000 and December 2005. Using time-averaged serum sodium (TA-Na) levels, we aimed to investigate whether hyponatremia can predict mortality in these patients.

**Results:**

Among the baseline parameters, serum sodium level was positively associated with serum albumin (β = 0.145; *p* = 0.003) and residual renal function (RRF) (β = 0.130; *p* = 0.018) and inversely associated with PD ultrafiltration (β = −0.114; *p* = 0.024) in a multivariable linear regression analysis. During a median follow-up of 34.8 months, 149 deaths were recorded. All-cause death occurred in 81 (55.9%) patients in the lowest tertile compared to 37 (25.0%) and 31 (20.9%) patients in the middle and highest tertiles, respectively. After adjusting for multiple potentially confounding covariates, increased TA-Na level was associated with a significantly decreased risk of all-cause (HR per 1 mEq/L increase, 0.79; 95% CI, 0.73–0.86; *p*<0.001) and infection-related (HR per 1 mEq/L increase, 0.77; 95% CI, 0.70–0.85; *p*<0.001) deaths.

**Conclusions:**

This study showed that hyponatremia is an independent predictor of mortality in PD patients. Nevertheless, whether correcting hyponatremia improves patient survival is unknown. Future interventional studies should address this question more appropriately.

## Introduction

Electrolyte handling is universally impaired in patients with chronic kidney disease (CKD) because of failing kidney function. Therefore, these patients are vulnerable to complications caused by electrolyte imbalance. Although this issue is partly resolved by dialysis treatment, patients with end-stage renal disease (ESRD) commonly suffer from electrolyte disturbances [1].

Hyponatremia, usually defined as a serum sodium concentration <135 mEq/L, is one of the most common electrolyte disorders and is believed to be an important risk factor for mortality in patients with many serious medical conditions including advanced heart failure or liver cirrhosis [2]–[5]. In these conditions, it is generally assumed that hyponatremia reflects the severity of the underlying disease rather than directly contributing to mortality. Hyponatremia is also common in CKD patients [1]. Moreover, recent studies have shown that lower serum sodium concentrations are associated with increased mortality in maintenance hemodialysis (HD) patients and CKD patients prior to dialysis therapy [6]–[10]. However, the underlying mechanisms are complex and still need to be clarified. In addition, it is still uncertain whether hyponatremia itself can contribute to mortality or merely represents a surrogate marker for other unknown risk factors in chronic maintenance dialysis patients.

Although peritoneal dialysis (PD) is an established therapeutic modality in ESRD, characteristics of dialysis treatment clearly differ between PD and HD. In particular, serum levels of electrolytes fluctuate between dialysis sessions in patients receiving HD due to the intermittent nature of HD treatment, while these levels remain relatively stable in PD patients. This unique feature led us to investigate which factors are associated with hyponatremia and its clinical outcomes in patients chronic PD. To date, few studies have addressed this issue, in particular the relationship between serum sodium level and risk of death, in PD patients [11]–[15]. Therefore, the purpose of this study was to investigate whether hyponatremia can predict mortality in a large prospective cohort of incident patients undergoing PD.

## Methods

### Ethics statement

The study was carried out in accordance with the Declaration of Helsinki and approved by the Institutional Review Board of Ilsan Hospital Clinical Trial Center. We obtained informed written consent from all participants involved in our study.

### Patients

**Figure 1:**
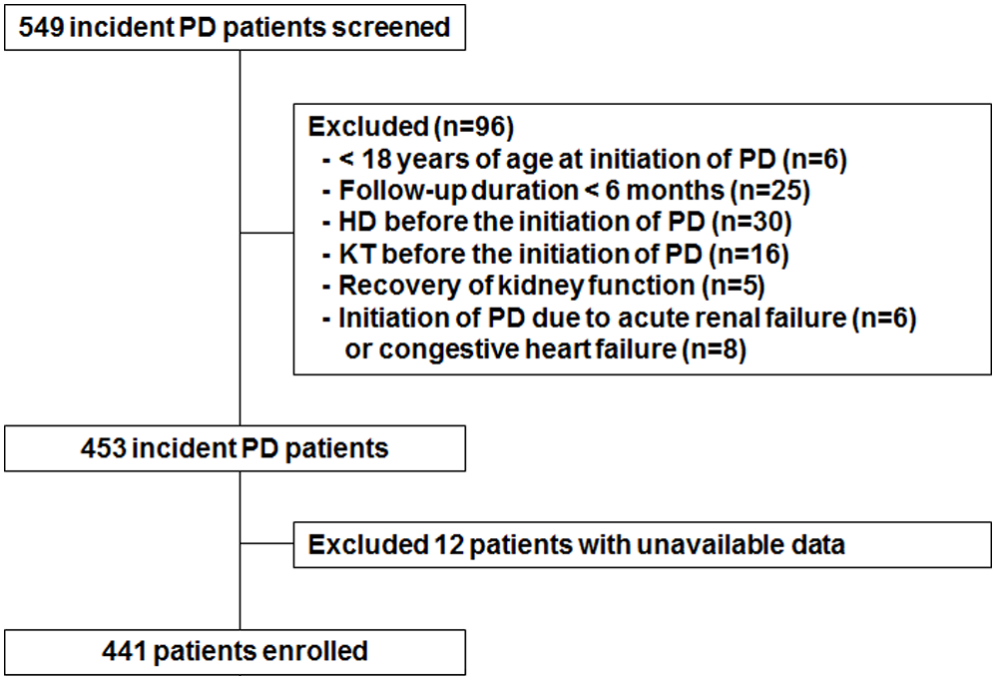
Flow chart of participants in the cohort. PD, peritoneal dialysis; HD, hemodialysis; KT, kidney transplant.

The study population included 549 ESRD patients who started PD at Yonsei University Severance Hospital or NHIS Ilsan Hospital between January 2000 and December 2005. Exclusion criteria were 1) <18 years of age at initiation of PD, 2) follow-up duration less than 6 months, 3) prior history of HD or a kidney transplant before the initiation of PD, 4) recovery of kidney function, or 5) initiation of PD for other reasons such as acute renal failure or congestive heart failure. Therefore, this prospective observational study included a total of 441 incident patients (Figure 1).

### Data collection

Demographic and clinical data were collected at the beginning of PD including age, gender, body mass index (BMI) calculated as weight/(height)^2^, cause of ESRD, and presence of diabetes and coronary artery disease. Each subject was scored using the Charlson Comorbidity Index at the start of PD. This index is a scoring system that includes weight factors for important concomitant diseases [16], [17]. All patients underwent urea kinetic studies within three months of PD initiation including Kt/V urea, normalized protein catabolic rate (nPCR), percentage of lean body mass (%LBM), PD ultrafiltration, and residual glomerular filtration rate (GFR). Residual GFR was calculated as the average urea and creatinine clearance from a 24-h urine collection [18]. nPCR was calculated by the methods described by Randerson et al. [19] and normalized to standard body weight (total body water/0.58). Total body water was estimated by Watson formula [20]. The %LBM was determined from creatinine kinetics according to Keshaviah et al. [21]. Laboratory data obtained at the time of dialysis adequacy measurement were considered baseline values and included serum sodium, potassium, and bicarbonate concentrations, serum albumin, ferritin, and serum C-reactive protein (CRP) levels. All laboratory parameters were recorded longitudinally throughout the follow-up period, and were calculated as an average of the mean of measurements every 3-month period. Because dialysis adequacy is generally measured within the first month after starting dialysis therapy and every 6 month thereafter in our centers, time-averaged dialysis adequacy parameters were determined as an average of the mean of the measurements in every 6-month period. Serum sodium concentration was measured by an electrode-based method (UniCel DXC 800; Beckman Coulter, Inc., CA, USA) and was corrected for serum glucose level (in such a case of serum glucose above a normal of 100 mg/dL) using the following formula: Corrected sodium = measured sodium+0.016×(serum glucose−100) [22].

### Study outcomes

Study participants were followed until December 31, 2011. The primary outcome parameters were all-cause, cardiovascular, and infection-related mortality.

### Statistical analysis

All values are expressed as the mean ± standard deviation or percentages. Statistical analyses were performed using SPSS for Windows version 13.0 (SPSS, Inc., Chicago, IL, USA) and the software packages R version 3.0.2. Data were analyzed using Student’s *t*–test and the Chi-square test, and ANOVA was used for multiple comparisons. The Kolmogorov-Smirnov test was used to determine the normality of the distribution of parameters. If data were not normally distributed, they were expressed as the median and interquartile range (or after log-transformation) and were compared using the Mann–Whitney test or Kruskal-Wallis test. Relationships between serum sodium and continuous variables were assessed using Pearson’s correlation coefficient, and categorical variables were evaluated using Spearman’s *R* test. Multiple linear regression analysis was performed to identify the determinants of serum sodium level.

Under conditions of competing risk, Kaplan-Meier analyses and traditional Cox regression models can produce misleading results, so corresponding competing risk methods were used [23]–[25]. The cumulative incidence of death was compared among 3 groups based on tertile of time-averaged serum sodium (TA-Na) levels (<137, 137 to 139, and ≥139 mEq/L) using the K-sample test developed by Gray [23]. Data for switch to HD, kidney transplantation, and loss to follow-up were censored in the analysis. Patients who died within the first 3 months after converting to HD or receiving a kidney graft were considered deaths related to PD. To determine risk factors for mortality, multivariate Cox regression using methods of Fine and Gray [24] was performed, and four different models were constructed; Model 1 adjusted for epidemiologic parameters including age, sex, BMI, and Charlson Comorbidity Index score. Model 2 adjusted for all parameters in model 1 plus medication use including dose of furosemide, use of icodextrin, and use of high-glucose dialysate. Model 3 adjusted for all parameters in model 2 plus dialysis dose including PD ultrafiltration and total Kt/Vurea. Model 4 adjusted for all parameters in model 3 plus malnutrition-inflammatory parameters including serum potassium, serum bicarbonate, serum albumin, serum ferritin, CRP, residual GFR, nPCR, and %LBM. The results are expressed as a hazard ratio (HR) and 95% confidence interval (CI). First-order interaction terms between covariates were examined for all models, but there was no evidence of an interaction between those covariates. A *p*–value less than 0.05 was considered statistically significant.

## Results

### Patient characteristics

The mean age of the patients was 59.2 years (range, 22 to 85 years), 54.4% were males, and patients were on PD for a mean duration of 43.2 months (range, 6 to 142 months). The mean baseline and TA-Na levels were 138.2±3.7 mEq/L and 137.7±2.7 mEq/L (median, 138.1 mEq/L; range, 126.2 to 143.1 mEq/L) respectively. Table 1 details the baseline characteristics of the 441 patients categorized by tertile of TA-Na level. Serum albumin (*p* for trend <0.001), residual GFR (*p* for trend = 0.004), nPCR (*p* for trend = 0.012), and %LBM (*p* for trend <0.001) were higher at higher TA-Na level, whereas PD ultrafiltration (*p* for trend = 0.008) and use of icodextrin (*p* for trend <0.001) were lower at higher TA-Na level. However, we observed no significant differences in the types of antihypertensive agents, dosage of furosemide used, or use of high-glucose dialysate according to TA-Na level.

**Table 1:** Clinical characteristics of the study subjects stratified by time-averaged serum sodium level.

	Time-averaged serum sodium (mEq/L; number of subjects)				
	Total(n = 441)	<137(n = 145)	137 to <139(n = 148)	≥139(n = 148)	*p* for trend
Age (years)	59.2±13.8	60.6±13.4	58.3±14.2	58.8±13.8	0.251
Gender (male)	240 (54.4)	76 (52.4)	79 (53.4)	85 (57.4)	0.388
Body massindex (kg/m^2^)	22.9±6.7	22.4±2.6	23.5±7.7	22.6±3.3	0.846
Presence ofdiabetes mellitus	227 (51.5)	73 (50.3)	73 (49.3)	81 (54.7)	0.446
Presence ofcoronary arterydisease	70 (15.9)	27 (18.6)	24 (16.2)	19 (12.8)	0.176
CharlsonComorbidityIndex score	3.0±1.0	3.2±1.0	3.0±1.1	3.0±0.9	0.065
Laboratoryfindings*					
Serumsodium (mEq/L)	137.7±2.7	134.5±2.1	138.0±0.6	140.3±0.9	<0.001
Serumpotassium(mEq/L)	4.1±0.7	4.2±0.8	4.1±0.6	4.1±0.7	0.274
Serumbicarbonate(mEq/L)	25.9±2.4	25.7±2.3	26.1±2.3	26.0±2.5	0.206
Serumalbumin (g/dL)	3.1±0.5	2.9±0.5	3.1±0.6	3.2±0.5	<0.001
Serumferritin (ng/mL)	274.3 [52–521]	294.7 [52–509]	258.3 [59–521]	271.2 [56–514]	0.314
C-reactive protein(mg/dL)	0.7 [0.01–16.2]	0.8 [0.01–14.2]	0.4 [0.01–6.3]	0.8 [0.01–16.2]	0.623
Residual GFR(mL/min/1.73m^2^)*	4.0 [0.2–29.3]	3.9 [0.2–18.9]	4.0 [0.3–29.3]	4.4 [0.3–19.0]	0.004
Dailyultrafiltration(mL)*					
Peritoneal dialysis	731.6±569.3	811.2±601.6	751.2±563.1	634.1±531.4	0.008
Total	1827.6±691.4	1847.6±706.0	1794.2±700.2	1841.4±671.3	0.943
Automatedperitoneal dialysis	5 (1.1)	1 (0.7)	2 (1.4)	2 (1.4)	0.594
Icodextrin usedaily	35 (7.9)	25 (17.2)	10 (6.8)	0 (0.0)	<0.001
2.5% and/or4.25% dialysateuse daily	105 (23.8)	38 (26.2)	41 (27.7)	26 (17.6)	0.081
Total weeklyKt/V urea*	2.3±0.8	2.2±0.7	2.3±0.9	2.4±0.8	0.061
nPCR (g/Kg/day)*	0.9±0.3	0.9±0.3	0.9±0.3	1.0±0.2	0.012
Lean body mass(% body weight)*	66.4±12.4	61.4±11.9	66.8±13.8	67.4±14.2	<0.001
Anti-hypertensivemedications					
ACE inhibitoror ARB	302 (68.5)	98 (67.6)	104 (70.3)	100 (67.6)	0.995
Alpha and/or beta blocker	217 (49.2)	72 (49.7)	78 (52.7)	67 (45.3)	0.450
Calciumchannel blocker	265 (60.1)	86 (59.3)	91 (61.5)	88 (59.5)	0.981
Thiazideor thiazide-likediuretics	48 (10.9)	19 (13.1)	13 (8.8)	16 (10.8)	0.534
Lasix orother loopdiuretics	200 (45.4)	64 (44.1)	71 (48.0)	65 (43.9)	0.966
Dose offurosemide(mg/day)	46.8±61.6	48.7±63.5	52.4±66.1	39.4±54.4	0.192

Values for categorical variables are given as number (percentage); values for continuous variables are given as mean ± standard deviation or median [interquartile range].

*Laboratory and dialysis-specific parameters are given as time-averaged values. GFR, glomerular filtration rate; nPCR, normalized protein catabolic rate; ACE, angiotensin converting enzyme; ARB, angiotensin receptor blocker.

### Factors associated with baseline serum sodium

Correlation analyses were performed to identify factors associated with baseline serum sodium level (Table 2). There was no correlation between age, gender, BMI, Charlson Comorbidity Index score, serum potassium, serum CRP, total Kt/V urea, nPCR and serum sodium. In contrast, baseline serum sodium level positively correlated with serum albumin (ρ = 0.177; *p*<0.001), residual GFR (ρ = 0.211; *p*<0.001), and %LBM (ρ = 0.109; *p* = 0.022), whereas it inversely correlated with serum ferritin level (ρ = −0.133; *p* = 0.005) and PD ultrafiltration (ρ = −0.169; *p*<0.001). A multivariate linear regression analysis that was adjusted for these factors revealed that serum albumin (β = 0.145; *p* = 0.003), residual GFR (β = 0.130; *p* = 0.018), and PD ultrafiltration (β = −0.114; *p* = 0.024) were independently associated with baseline serum sodium.

**Table 2:** Cross-sectional correlation analyses between baseline serum sodium level and patient characteristics.

		Serum Na	Age	BMI	CCI	K	Albumin	Ferritin*	CRP*	Residual GFR*	PD UF	TotalKt/Vurea	nPCR	LBM
Serum Na	ρ	1	−0.047	0.072	−0.058	0.022	0.177	−0.133	0.006	0.211	−0.169	0.068	0.054	0.109
	*p*	-	0.329	0.129	0.223	0.640	<0.001	0.005	0.909	<0.001	<0.001	0.156	0.260	0.022
Age	ρ		1	−0.019	0.041	−0.133	−0.230	0.266	0.261	−0.058	−0.026	0.033	−0.165	−0.458
	*p*		−	0.695	0.695	0.005	<0.001	<0.001	<0.001	0.222	0.590	0.489	<0.001	<0.001
BMI	ρ			1	0.083	−0.007	0.021	0.024	−0.070	−0.039	0.026	−0.013	0.227	−0.046
	*p*			−	0.082	0.880	0.661	0.609	0.161	0.419	0.588	0.780	<0.001	0.333
CCI	ρ				1	0.021	−0.058	0.032	0.030	−0.044	0.126	−0.019	−0.020	0.021
	*p*				−	0.659	0.226	0.508	0.542	0.360	0.008	0.697	0.675	0.660
K	ρ					1	0.031	−0.140	−0.070	−0.003	0.121	−0.074	0.152	0.098
	*p*					-	0.510	0.003	0.162	0.958	0.574	<0.001	0.001	0.039
Albumin	ρ						1	-0.094	−0.096	0.145	−0.015	0.147	0.271	0.269
	*p*						-	0.048	0.053	0.002	0.754	0.002	<0.001	<0.001
Ferritin*	ρ							1	0.243	−0.246	0.129	−0.088	−0.152	−0.218
	*p*							-	<0.001	<0.001	0.007	0.066	0.001	<0.001
CRP*	ρ								1	-0.070	0.096	0.047	−0.114	−0.193
	*p*								-	0.158	0.055	0.342	0.022	<0.001
Residual GFR*	ρ									1	−0.365	0.533	0.238	0.376
	*p*									-	<0.001	<0.001	<0.001	<0.001
PD UF	ρ										1	0.065	0.067	0.027
	*p*										-	0.171	0.157	0.577
Total Kt/Vurea	ρ											1	0.451	0.293
	*p*											-	<0.001	<0.001
nPCR	ρ												1	0.464
	*p*												-	<0.001
LBM	ρ													1
	*p*													-

*Data for ferritin, CRP, and residual GFR were log-transformed.

BMI, body mass index; CCI, Charlson Comorbidity Index score; Na, sodium; K, serum potassium; tCO2, serum bicarbonate; CRP, C-reactive protein; GFR, glomerular filtration rate; PD UF, peritoneal dialysis ultrafiltration; nPCR, normalized protein, LBM, percentage of lean body mass.

### Hyponatremia as a predictor of mortality

During follow-up, 149 deaths were recorded, and the median survival period was 34.8 months (range, 6.0 to 142.2 months). Infection (37.6%) was the most common cause of death in this study, followed by cardiovascular disease (36.2%). All-cause death occurred in 81 (55.9%) patients in the lowest tertile compared with 37 (25.0%) and 31 (20.9%) patients in the middle and highest tertiles (*p*<0.001), respectively. Similar findings were observed for cardiovascular and infection-related deaths (data not shown).

**Figure 2:**
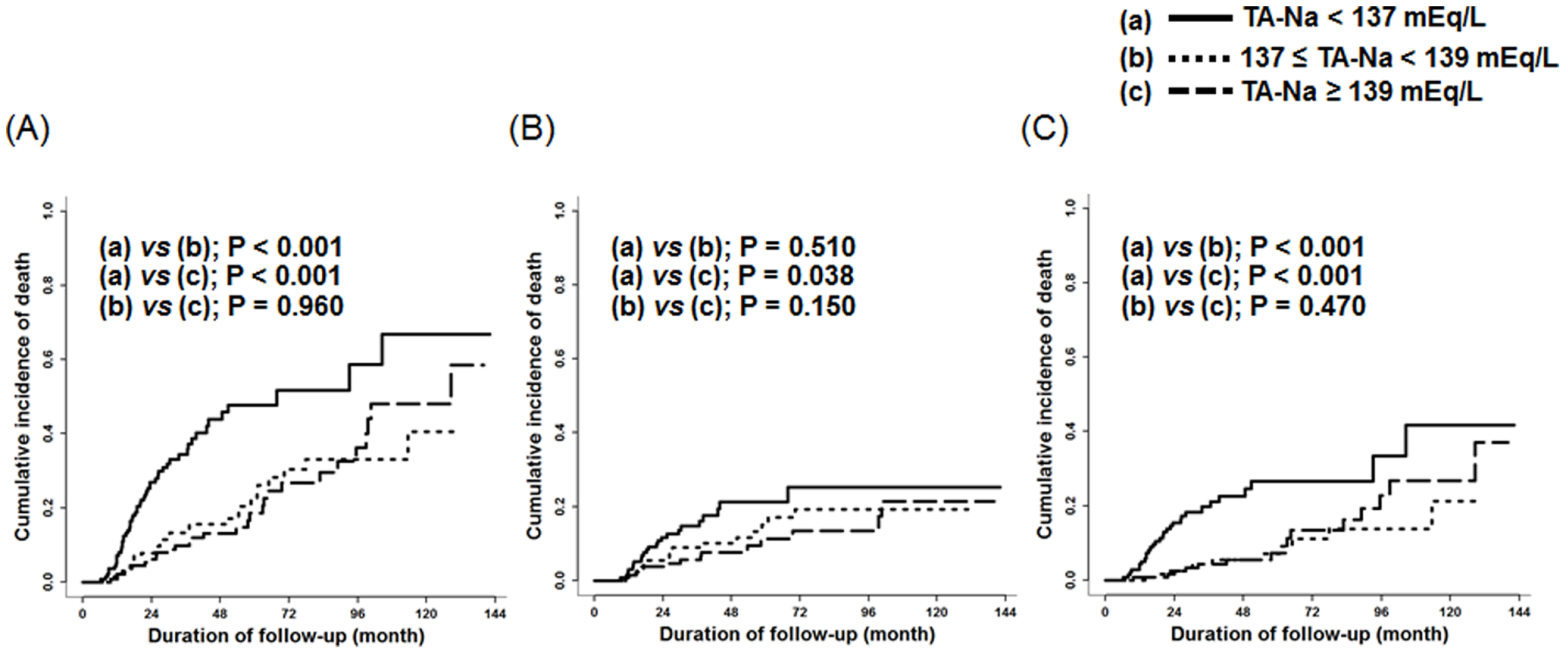
The Cumulative incidence of all-cause (A), cardiovascular (B), and infection-related (C) mortality based on the level of time-averaged serum sodium (TA-Na).

Higher TA-Na was associated with lower mortality (Table 3). Even after adjusting for various parameters, the association between the two was significant and consistent. Using serum TA-Na as a continuous variable, the HR for all-cause mortality was 0.79 for every 1 mEq/L increase in TA-Na (95% CI, 0.73–0.86; *p*<0.001), indicating that higher TA-Na was significantly associated with decreased risk of mortality. Moreover, the increased mortality risk associated with lower TA-Na was similar for infection-related (HR, 0.77 per 1 mEq/L higher TA-Na; 95% CI, 0.70–0.85; *p*<0.001) death. In addition, patients with TA-Na<137 mEq/L conferred a 3.35- and 3.18-fold increased risk of all-cause and infection-related mortality, respectively, compared with patients with TA-Na≥139 mEq/L. However, the risk for cardiovascular death was not associated with TA-NA levels. The cumulative incidence of death was significantly lower in patients with higher TA-Na level compared to patients with a TA-Na level <137 mEq/L (Figure 2).

**Table 3:** Multivariable Cox regression analyses for all-cause, cardiovascular, and infection-related mortality.

	Model 1			Model 2			Model 3			Model 4		
	HR	(95% CI)	*p*	HR	(95% CI)	*p*	HR	(95% CI)	*p*	HR	(95% CI)	*p*
**Continuous model**												
(per 1mEq/L TA-Na increase)												
All-cause mortality	0.78	(0.73–0.84)	<0.001	0.77	(0.72–0.83)	<0.001	0.78	(0.73–0.84)	<0.001	0.79	(0.73–0.86)	<0.001
Cardiovascular mortality	0.93	(0.84–1.02)	0.100	0.91	(0.82–1.00)	0.043	0.93	(0.84–1.02)	0.120	0.95	(0.84–1.08)	0.440
Infection-related mortality	0.78	(0.72–0.85)	<0.001	0.78	(0.72–0.85)	<0.001	0.78	(0.71–0.85)	<0.001	0.77	(0.70–0.85)	<0.001
**Categorical (tertile) model**												
All-cause mortality												
TA-Na<137 mEq/L	3.35	(2.15–5.24)	<0.001	3.70	(2.34–5.85)	<0.001	3.53	(2.23–5.60)	<0.001	3.35	(2.01–5.60)	<0.001
137 mEq/L≤TA-Na<139 mEq/L	1.13	(0.67–1.91)	0.640	1.16	(0.69–1.96)	0.570	1.12	(0.67–1.88)	0.660	1.17	(0.66–2.07)	0.600
TA-Na≥139 mEq/L	1.00	(reference)		1.00	(reference)		1.00	(reference)		1.00	(reference)	
Cardiovascular mortality												
TA-Na<137 mEq/L	1.82	(0.93–3.55)	0.079	2.09	(1.06–4.10)	0.033	1.90	(0.96–3.77)	0.067	1.70	(0.78–3.71)	0.180
137 mEq/L≤TA-Na<139 mEq/L	1.29	(0.64–2.61)	0.480	1.29	(0.63–2.61)	0.480	1.20	(0.59–2.45)	0.610	1.37	(0.64–2.92)	0.420
TA-Na≥139 mEq/L	1.00	(reference)		1.00	(reference)		1.00	(reference)		1.00	(reference)	
Infection-related mortality												
TA-Na<137 mEq/L	2.94	(1.65–5.26)	<0.001	3.05	(1.68–5.51)	<0.001	2.94	(1.60–5.41)	<0.001	3.18	(1.58–6.41)	0.0012
137 mEq/L≤TA-Na<139 mEq/L	0.79	(0.36–1.74)	0.560	0.81	(0.36–1.76)	0.570	0.79	(0.36–1.76)	0.570	0.78	(0.31–2.02)	0.610
TA-Na≥139 mEq/L	1.00	(reference)		1.00	(reference)		1.00	(reference)		1.00	(reference)	

TA-Na, time-averaged serum sodium; HR, hazard ratio; CI, confidence interval. Model 1 adjusted for epidemiologic parameters including age, sex, body mass index, and Charlson Comorbidity Index score. Model 2 adjusted for all model 1 parameters plus medication including dose of furosemide, use of icodextrin, and use of high glucose dialysate. Model 3 adjusted for all model 2 parameters plus dialysis dose including peritoneal dialysis ultrafiltration (PDUF) and total Kt/V urea. Model 4 adjusted for all model 3 parameters plus malnutrition-inflammatory parameters including serum potassium, serum bicarbonate, serum albumin, serum ferritin, C-reactive protein (CRP), residual glomerular filtration rate (GFR), normalized protein catabolic rate (nPCR), and percentage of lean body mass (%LBM). Laboratory (serum sodium, potassium, bicarbonate, albumin, ferritin, and CRP) and dialysis-specific (PDUF, Kt/V urea, residual GFR, nPCR, %LBM) parameters are given as time-averaged values.

## Discussion

In this study, we sought to delineate the relationship between serum sodium level and mortality in our ESRD cohort of patients with PD. We showed that serum sodium level exhibited a significant positive association with serum albumin and residual renal function (RRF), while it inversely correlated with PD ultrafiltration. In addition, low TA-Na level independently predicted all-cause mortality, suggesting that hyponatremia is a potential predictor of adverse outcomes in these patients.

Serum sodium concentration in humans is tightly regulated and the presence of hypotonic hyponatremia implies that the extracellular fluid compartment contains water in excess of sodium. Interestingly, the kidneys’ ability to dilute urine, that is to elaborate urine with minimal osmolality, is generally preserved even in advanced CKD. However, its ability to concentrate is, indeed, severely limited in these patients [26]. Furthermore, in ESRD patients, water and sodium removal are almost exclusively determined by dialysis procedure, particularly when accompanied by complete loss of RRF. Therefore, dialysis-dependent patients are vulnerable to develop dysnatremia. However, the clinical impact of hyponatremia in these patients has been inadequately studied.

In our study, hyponatremia (defined as <135 mEq/L) was present in 58 patients (13.2%) at the initiation of PD. However, previous studies of PD patients have used a more strict definition of hyponatremia (defined as ≤130 mEq/L on 2 consecutive measurements) [13]–[15]. When we use this definition, 11 patients (2.5%) presented with hyponatremia at the start of PD. In addition, 21 patients (4.8%) developed hyponatremia during follow-up, which was comparable with the 5.2% of patients reported in a previous study by Zevallos *et al.* [13] but much lower than the 14.5% of patients observed in a recent study by Dimitriadis *et al* [14]. However, when we defined hyponatremia as <135 mEq/L, which is the threshold generally accepted in clinical practice, 115 patients (26.1%) developed hyponatremia during the follow-up period. These findings suggest that hyponatremia is common in PD patients, although the incidence may differ depending on the chosen definition.

The underlying mechanisms for hyponatremia in PD patients are complex and unclear. In general, as suggested by Edelman *et al.* [27], serum sodium concentration is determined by rapidly exchangeable body sodium, potassium, and total body water. Thus, hypotonic hyponatremia typically develops due to decrease in the fraction of body sodium and potassium over body water. Such reduction is potentially caused by various combinations of changes in body cations and water. In particular, the cation fraction can be often decreased in patients on dialysis because these patients are prone to fluid overload. In addition to this mechanism, dialysis-specific conditions should be taken into to explain hyponatremia in dialysis patients. Of note, therapeutic modality itself such as diffusive transport, convective transport, and ultrafiltration can induce the changes of body cations and water and thus results in altered serum sodium concentrations in PD patients [28]. Therefore, in patients on PD, alterations in serum sodium concentrations can occur as a result of the dialytic and nondialytic changes in the mass balance of body sodium, body potassium, and body water.

In addition, osmotic shift of water from the intracellular into the extracellular compartment and intracellular entry of sodium are putative mechanisms of PD-associated hyponatremia in certain clinical states, including potassium deficit, protein-energy wasting (PEW) and use of icodextrin solutions as PD dialysate [13], [29]–[31]. However, although there is evidence suggesting that entry of extracellular sodium into the cells causes hyponatremia in various settings [13], [29]–[31], hyponatremia is unlikely to occur unless such a shift is accompanied by imbalances of monovalent cations and water that decrease the cation fraction as aforementioned.

The most important finding in this study is the strong and significant association between lower serum sodium concentration and mortality in PD patients. This association has previously been shown in maintenance dialysis patients and CKD patients prior to dialysis therapy [6]–[10], [12], [15]. In keeping with these, our robust finding supports evidence that hyponatremia portends a poor prognosis in these patients. In multivariate models with rigorous adjustments, low serum sodium concentration remained an independent predictor of mortality. In addition, patients with TA-Na level <137 mEq/L had adjusted HRs for all-cause, and infection-related mortality of 3.35 and 3.18, respectively, compared to the reference group with TA-Na level ≥139 mEq/L, indicating that higher serum sodium level is associated significantly with lower mortality risk. Relevant to our finding is a recent observation in HD patients based on data from the Dialysis Outcomes and Practice Pattern Study, showing that patients with mean serum sodium level <137 mEq/L had a 45% higher risk of death compared with patients with serum sodium ≥140 mEq/L [7].

The underlying mechanisms for increased mortality in advanced CKD patients with lower serum sodium level are also unclear. In severely ill patients without CKD, vasopressin is secreted in response to neurohormonal activation. Whether vasopressin contributes to the development of hyponatremia in patients with CKD is largely unknown. Possibly, vasopressin could explain the association between hyponatremia and mortality in this population if it acts in the same manner as in non-CKD patients with serious underlying conditions [32]. In addition, hyponatremia is related to other adverse features. In particular, reduced RRF may limit free water clearance, resulting in hyponatremia. Interestingly, RRF was identified as a significant factor associated with baseline serum sodium in this study; higher residual GFR was independently associated with a higher serum sodium concentration. This finding suggests that residual kidney function even in the advanced stage can contribute to sodium handling and prevent hyponatremia by excreting relatively more water than sodium. Conversely, loss of RRF in the end may result in inability to control excess water, leading to hyponatremia. Furthermore, PEW status may affect sodium level by depleting intracellular potassium and solutes [13], [29]. In the present study, there were significant associations between serum sodium level and some nutritional markers such as serum albumin and %LBM. If this is the case, low sodium level can represent underlying unfavorable conditions. Finally, inflammatory response has recently been suggested as a cause of hyponatremia [33]–[35]. Among many inflammatory markers, interleukin-6 is reported to induce central vasopressin secretion [35]. Of note, loss of RRF, PEW, and inflammation are established predictors of mortality in patients on dialysis [36]–[38], and all of the aforementioned mechanisms together may account for the association between hyponatremia and mortality in these patients.

There are several limitations to the present study. This was an observational study with a relatively small sample size. Hence, causality of our findings needs further confirmation. In particular, there has been much concern about whether hyponatremia directly causes death or is merely a marker of disease severity. Furthermore, it is unknown whether correcting hyponatremia can improve clinical outcomes in CKD patients. A detailed discussion about these issues is beyond our scope. However, it is likely that hyponatremia can occur in severe underlying conditions and may itself further increase the risk of adverse outcomes. Lack of information to further explain hyponatremia is another drawback. Because this was an observational study, we could not collect data describing the amount of sodium removed by PD or the amount being ingested in the diet. Other data representing overall volume status such as body weight changes, dietary water intake, and a bioelectrical impedance analysis were not available for analysis. Despite these limitations, this study showed that low TA-Na level independently predicted all-cause and infection-related mortality, even after extensive adjusting for demographic, clinical, laboratory, and dialysis-specific covariates. Our robust findings suggest that hyponatremia portends a poor prognosis, and thus physicians should be more meticulous in clinical practice with respect to correcting both this electrolyte imbalance and underlying disease.

## Conclusion

This study showed that a low serum sodium concentration was an independent predictor of mortality in PD patients. The relationship between low serum sodium level and adverse outcomes may be attributed to loss of RRF and underlying conditions such as PEW. Whether correcting hyponatremia improves patient survival requires further investigations.
